# The association between physical fitness and mental health among college students: a cross-sectional study

**DOI:** 10.3389/fpubh.2024.1384035

**Published:** 2024-07-16

**Authors:** Yu Hong, Jiaqi Shen, Yue Hu, Yuxuan Gu, Zhongjiang Bai, Yu Chen, Siyi Huang

**Affiliations:** ^1^Sports and Health Management, Changzhou Vocational Institute of Engineering, Changzhou Jiangsu, China; ^2^Department of Clinical Medicine, School of Medicine, Zhejiang University, Hangzhou, Zhejiang, China; ^3^Department of Oral Medicine, School of Medicine, Zhejiang University, Hangzhou, Zhejiang, China; ^4^Department of Social Security, Nanjing Normal University, Nanjing, Jiangsu, China; ^5^Department of Sports Science, College of Education, Zhejiang University, Hangzhou, Zhejiang, China; ^6^Department of Infectious Diseases and Public Health, City University of Hong Kong, Hong Kong, Hong Kong SAR, China

**Keywords:** physical fitness, mental health, Chinese college students, cross-sectional study, wellbeing of the youth

## Abstract

**Introduction:**

Physical inactivity and mental health disorders are emerging threats to public health in China. Previous research on links between exercise and mental wellbeing have focused on clinical populations, with little evidence from the general population, particularly younger adults. We aimed to investigate associations between physical fitness and mental health in Chinese college students.

**Methods:**

In this series, of cross-sectional observational studies, we enrolled 7,468 Chinese college students aged 16–24 years in 199 classes at Changzhou Vocational Institute of Engineering between Sept 1, 2017, and Jun 30, 2018. Exposures of interest were the students’ physical fitness level, measured by the Chinese University Students Physique Test. The primary outcomes were mental health situations, measured by the University Personality Inventory (UPI). The multivariable linear regression models were used to assess the relationship between the levels of physical fitness and mental disorders symptoms.

**Results:**

Completed mental health and physical fitness data were available for 6,724 participants aged 16–24 years. Compared with the failed group, the corresponding levels of difference in overall UPI scores were − 1.45 scores (95% CI: −2.45, −0.46; *p* < 0.01) for the passed group and − 2.95 scores (95% CI: −4.13, −1.77; p < 0.01) for the good group. Similar results were observed in the four different aspects of psychotic disorder symptoms.

**Discussion:**

There was a significantly negative correlation between the level of physical fitness and phycological situations among Chinese college students. Our findings highlight the psychological situations of students with low physical fitness levels should be concerned.

## Introduction

The marked decline in physical fitness among Chinese college students warrants significant concern. The 2020 Physical Fitness and Health Surveillance Report indicates that merely a third of students meet established physical standards, with obesity affecting 5.5% (1) of this population. This diminishing fitness trend is increasingly viewed as a factor contributing to greater susceptibility to infectious diseases, and individuals with lower fitness levels are more likely to struggle in coping with social challenges (2). Research suggests that improvements in physical fitness correlate with positive shifts in cardiovascular risk factors. Furthermore, there is a strong, inverse relationship between changes in physical fitness and long-term mortality rates, especially in the cardiovascular and all-cause mortality (3). Additionally, individuals with inadequate fitness often report higher instances of absenteeism from work or academic settings, imposing significant health and economic burdens on their families and the broader society (4). This scenario highlights the pressing need to address the decline in physical fitness and mitigate its comprehensive impact on public health and economic stability.

Mental health disorders present an additional challenge, with global prevalence figures for anxiety (301 million), major depression (280 million), and bipolar disorder (40 million) reaching alarming heights (5). Within China, mental disorders, excluding dementia, affect 9.3% of the population, encompassing conditions like anxiety, alcohol dependence, and schizophrenia (6). A particular study in 2020 identified a prevalence of 28.4% for depression among Chinese students (7). The financial impact of these disorders is non-negligible, equating to almost 5% of China’s gross domestic income, attributable to factors such as absenteeism, productivity loss, and increased healthcare expenditures (8). Furthermore, a meta-analysis has highlighted that the risk of all-cause mortality is more than two-fold higher in individuals with psychological disorders compared to their healthier counterparts (9). The interplay between declining physical fitness and mental health concerns is emerging as a significant public health crisis in China.

The interconnection between physical and mental health has been well-documented within patient populations. For instance, individuals with type 2 diabetes are at a doubled risk for depression relative to the general population. Studies have shown that almost half of the patients with cancer experience concurrent mental health issues, and addressing depressive symptoms may prolong their survival (10). Evidence also points to increased susceptibility to diseases like cancer, type 2 diabetes, cardiovascular disease, and asthma among those with severe mental illnesses (11). A longitudinal study covering 15 cohorts observed that psychiatric patients on medication exhibit diminished physical activity levels, correlating directly with the dosage of medication (12). While past research has largely centered on patient cohorts, studies focusing on the general populace, particularly the young adult demographic, are sparse. The definitive relationship between physical fitness and mental health within the broader population remains to be elucidated.

In an effort to bridge this knowledge gap, our study conducted a cross-sectional evaluation of the link between physical fitness levels and mental health among Chinese college students. In addition, the situation of physical fitness could be changed through the regular physical exercise, thus, exploring the association between the levels of physical fitness and mental health might provide the essential evidence for the psychological disorders prevention, especially in the general population.

## Materials and methods

### Data sources

We used a series, of cross-sectional datasets of freshmen enrollment surveys and scores of the Students Physique Test initiated in 2017 and 2018. The freshmen enrollment psychological surveys were conducted at Changzhou Vocational Institute of Engineering. The psychological surveys were used to assess the profile of college students’ psychological situations. The student’s physical fitness status was measured by trained staff in Chinese University Students’ Physique Tests. All participants were typical college entrants who underwent comprehensive medical and psychological evaluations at the time of their university admission. The data pertaining to mental health, which form a crucial part of our study, were derived from these initial screenings. Prior to these examinations, none of the participants had reported any psychological disorders or other physiological impairments.

Our study was designed to utilize a multistage stratified, cluster-randomized, college-based, sampling design. The final eligible participants were Chinese college students aged 16–24 years from Changzhou Vocational Institute of Engineering. We excluded participants who were unable to finish the Physique Test or refused ethical informed consent. After excluding 744 participants with missing data on exposures and outcomes, a total of 6,724 participants recruited from 199 classes were included in the final analysis.

### Ethics statement

The data were collected from June to October 2018, and this study was approved by the Medical Ethics Committee, Department of Psychological and Behavioral Sciences, Zhejiang University (approval number: 2022060). The study was carried out under the ethical guidelines of the Department of Psychological and Behavioral Sciences, Zhejiang University. Before we conducted this survey, informed written consent was given by the participants or their guardians. Consent was given by parents or caregivers for participants under the age of 18 under the above ethical guidelines. Moreover, all participants’ personal information remained confidential.

### Measurements of mental health situations

University Personality Inventory (UPI) was used to assess college students’ mental health conditions, covering physical symptoms, schizophrenia, depression and neuroticism, and persecutory beliefs aspects. UPI is a widely validated mental health instrument for screening the psychological situations of college students in China (13). UPI tests were just delivered online. When students enrolled in our investigations, they would be taken into the computer room to finish UPI tests by trained staff. In the UPI, only 56 items should be analyzed, because there were 4 items (5, 20, 35, and 50) being excluded, and treated as pseudo measurements. The 56 items are left as a multidimensional instrument for screening college students’ mental health situations, and a score of 1 was given for “yes,” and 0 was given for “no.” UPI scores from 0 to 56 and is a dimensional measure of mental health for university students. The higher scores in UPI represent the poorer the psychological situations.

### Measurements of physical fitness

The exposure of interest was the level of physical fitness, which was measured through the Chinese University Students’ Physique Test (14). Chinese University Students Physique Test was the validated instructions for monitoring students’ physical fitness, consisting of standing long jump (reflecting lower limb strength), 60-s sit-ups for female students (reflecting core strength), pull-ups for male students (reflecting upper limb and core strength), sit and reach (reflecting flexibility), 50-m dash (reflecting speed), and 800/1,000-m run test (reflecting cardiorespiratory fitness). Considering the sex differences, the context of physique fitness tests for males was slightly different from those for females. For example, females were asked to finish an 800-m run test to reflect cardiorespiratory fitness and 60-s sit-up tests, while males needed to finish a 1,000-m run test and pull-up tests (15). The specific process of physical fitness and details of measurements were illustrated in the Notice of the Ministry of Education on the National Student Physical Fitness Standard (14), published in 2014. The final score of the Chinese University Students Physique Test indicated the fitness level of the individual. The higher participants scored, the better their physical fitness was. Scores of 80 or above (out of 100) mean that the grade is “Good,” 60.0 ~ 79.9 is “Passed,” and, for scores below 60, the grade is “Failed.”

### Assessment of covariates

As part of the students’ physique test, several anthropometric data were obtained, covering weight, height, and eye vision. Body weight was measured to the closest 0.1 kg by digital scales, without shoes and heavy clothing, and the height was measured at 0.1 cm. Body Mass Index (BMI) was calculated [BMI = weight (kg)/height (m^2^)]. For the group under 18, the criteria of BMI categories were based on the age-and gender-specific cut-off points for overweight and obesity [Screening for overweight and obesity among school-age children and adolescents (WS/T 586—2017) and Screening standard for malnutrition of school-age children and adolescents (WS/T 456—2014)]. For the group aged 18 years old and above, the overweight and obesity metric is based on Chinese national surveys, suggesting that overweight was defined as BMI ≥ 24 kg/m^2^, and obesity was defined as BMI ≥ 28 kg/m^2^ (16). The eye vision was measured by trained medical staff with a logarithmic visual acuity chart. Age at baseline, vision, and BMI were collected as the continuous variable. Other potential categorical variables included survey year (2018 or 2019), major (Economics and Management, Sports and health management, Art and design, Intelligent Manufacturing, Chemical and Pharmaceutical Engineering, Architectural engineering, Inspection, Testing, and Certification), and gender (male or female), collected as the categorical variables.

### Statistical analysis

Descriptive analysis was described as frequencies (percentage) and mean (standard deviations, SD). We offered the descriptive summaries and assessed any potential differences between different groups by using χ^2^-tests for categorical variables and t-tests for continuous variables.

In the primary analyses, we utilized linear regression models to estimate the beta of the levels of physical fitness with UPI total scores, physical symptoms, schizophrenia, depression and neuroticism, and persecutory beliefs scores. Model 1 was adjusted for age and gender to isolate the direct effects of these fundamental demographic factors on mental health outcomes, acknowledging their established roles in epidemiological research. Model 2 was based on Model 1, additionally adjusted for survey year, college, BMI categories, and vision. Model 2, as a fully adjusted model, mitigated the influence of extraneous factors and enabling a more robust exploration of the association between physical fitness and mental health. Based on the fully adjusted model (Model 2), situations among different groups were illustrated via forest plots. Pairwise comparison in physical fitness levels between different groups used the Bonferroni method to correct the *p*-value.

We conducted a further sensitivity analysis and stratified analyses to assess the robustness of our primary findings. In our sensitivity analysis, we assessed the association scores of physical fitness with UPI total scores and each single mental health symptom score, and the scores of physical fitness were treated as the continuous variable. Accordingly, we have conducted stratified analyses based on gender. Statistical analyses were completed by SAS 9.4 (SAS Institute Inc., NC) and R (Version 4.0.3). We reported two-sided *p*-values and 95% confidence intervals (CIs) throughout, and a *p*-value <0.05 was considered an indicator of statistical significance.

## Results

### Characters of the study population

Among 6,724 participants aged 16–24 years, 67.0% were male (*n* = 4,503). The mean (SD) mental health score was 12.62(10.13) and the mean (SD) total score of fitness was 71.47(8.98). The proportion of Good, Pass, and Fail levels was 15.6, 76.5, and 7.9%, respectively. The points of initial sleep, sleep maintenance, and early morning awakening were 1.4(0.7), 1.5(0.7), and 1.5(0.8) respectively. As shown in Table 1, the participants with good physical fitness level were more likely to have better eye vision, come from the School of Sports and Health Management, and stay at a lower BMI.

**Table 1:** Demographic and health characteristics of eligible participants.

Characteristic		Overall (*n* = 6,724)	Fail (*n* = 532)	Good (*n* = 1,050)	Pass (*n* = 5,142)	*p*-value
Age, mean (SD)		18.3 (0.8)	18.2 (0.7)	18.4 (0.8)	18.3 (0.7)	<0.01
Gender, %						<0.01
	Female	2,221 (33.0)	71 (13.3)	460 (43.8)	1,690 (32.9)	
	Male	4,503 (67.0)	461 (86.7)	590 (56.2)	3,452 (67.1)	
BMI [mean (SD)]		22.3 (3.9)	28.1 (5.6)	20.7(2.0)	22.0(3.4)	<0.01
Vision [mean (SD)]		4.5 (0.4)	4.4 (0.4)	4.6 (0.4)	4.5(0.4)	<0.01
Survey year, %						0.05
	2017	3 (0.0)	0 (0.0)	0 (0.0)	3 (0.1)	
	2018	3,429 (51.0)	244 (45.9)	564 (53.7)	2,621 (51.0)	
	2019	3,292 (49.0)	288 (54.1)	486 (46.3)	2,518 (49.0)	
Physical fitness score [mean (SD)]		71.5 (9.0)	52.6(6.9)	84.1 (3.6)	70.8 (5.3)	<0.01
Score of mental health [mean (SD)]		12.6 (10.1)	13.5 (10.5)	11.5 (9.1)	12.8 (10.3)	<0.01
Somatization symptom [mean (SD)]		2.7 (2.7)	2.8 (2.7)	2.6 (2.6)	2.7 (2.7)	0.16
Schizophrenia symptom [mean (SD)]		4.0 (3.8)	4.4 (4.1)	3.6 (3.5)	4.0 (3.9)	<0.01
Depression [mean (SD)]		2.6 (2.6)	2.9 (2.7)	2.2 (2.4)	2.6 (2.7)	<0.01
Neuroticism and persecutory beliefs [mean (SD)]		4.8 (3.7)	5.0 (3.9)	4.5 (3.5)	4.9 (3.8)	<0.01
College (%)						<0.01
Chemical and pharmaceutical engineering		1,185 (17.6)	91 (17.1)	184 (17.5)	910 (17.7)	
Intelligent manufacturing		1722 (25.6)	199 (37.4)	218 (20.8)	1,305 (25.4)	
Architectural engineering		1,338 (19.9)	112 (21.1)	181 (17.2)	1,045 (20.3)	
Inspection, testing, and certification		734 (10.9)	47 (8.8)	121 (11.5)	566 (11.0)	
School of economics and management		814 (12.1)	30 (5.6)	136 (13.0)	648 (12.6)	
Art and design		658 (9.8)	44 (8.3)	83 (7.9)	531 (10.3)	
Sports and health management		273 (4.1)	9 (1.7)	127 (12.1)	137 (2.7)	

Age, body mass index (BMI), fitness score, mental health score and the sleep quality score were expressed as Mean (SD). Gender, Survey year, colleges were expressed as counts (percentages).

### Association between physical fitness level and UPI scores

**Figure 1:**
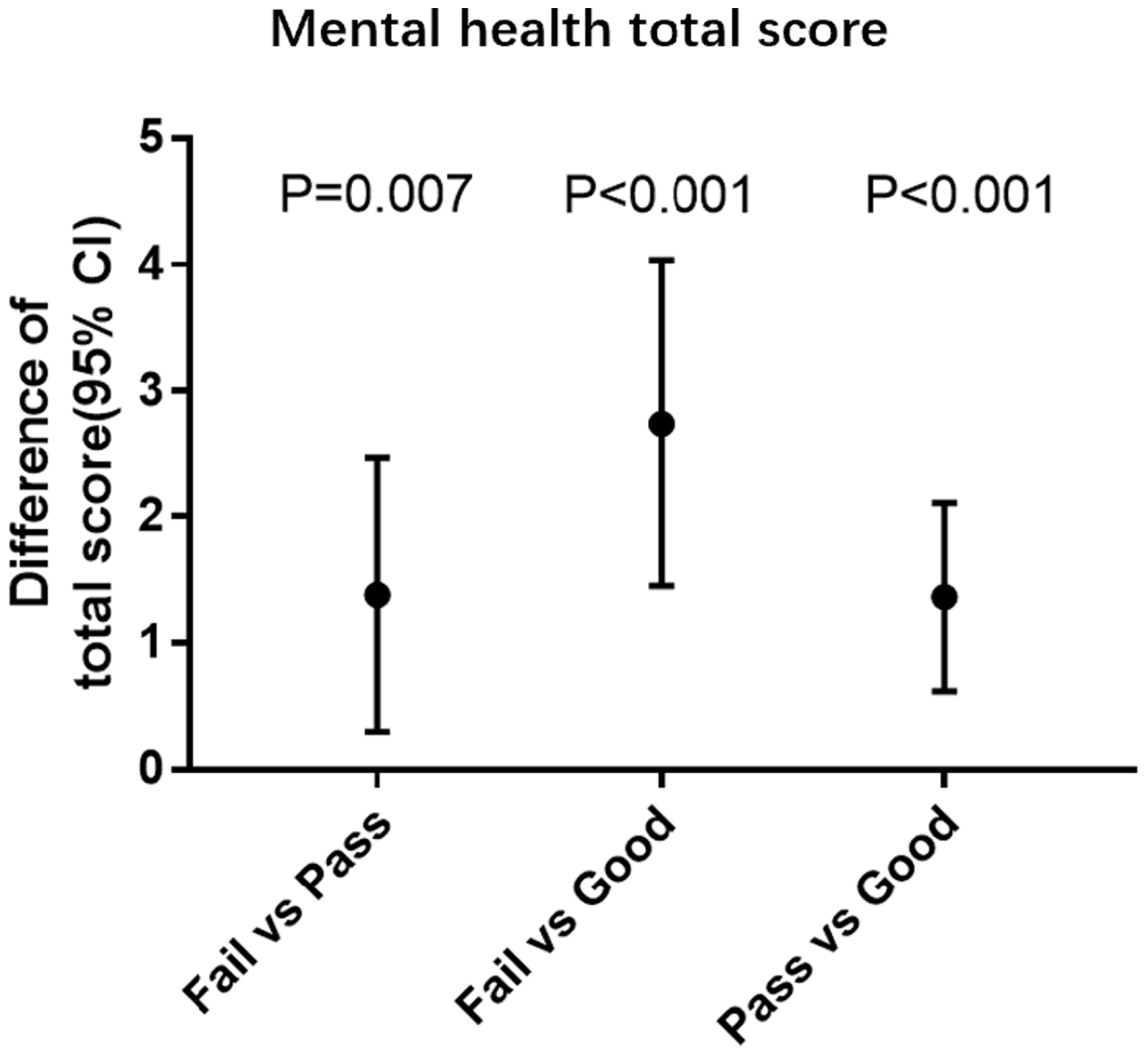
The association between mental health and physical fitness levels. Age at baseline, gender, survey year, college, BMI, and vision were adjusted. CI Confidence interval, P Significance level.

In the multivariable models adjusting for demographic information, survey year, college, BMI, and vision, participants with lower physical fitness levels had higher total scores of UPI (Table 2). Compared with the failed group, the corresponding levels of difference in overall UPI scores were − 1.45 scores (95% CI: −2.45, −0.46; *p* < 0.01) for the passed group and − 2.95 scores (95% CI: −4.13, −1.77; *p* < 0.01) for a good group. To help interpret the negative association, in the sensitivity analysis, we found one score increasing in physical fitness test was associated with 0.11 score (95%CI: −0.14 to −0.07, *p* < 0.01) decrease (Supplementary Table S1). The significant difference pairs of fail vs. pass, fail vs. good, and pass vs. good were illustrated in Figure 1.

**Table 2:** Linear regression results of the levels of physical fitness and UPI scores among the college students.

Levels of physical fitness	No. of participants	Model 1^a^		Model 2^b^	
		β (95%CI)	*p*-value	β (95%CI)	*p*-value
Failed	532	Reference		Reference	
Passed	5,142	−1.26(−2.17, −0.36)	<0.01	−1.45(−2.45, −0.46)	<0.01
Good	1,050	−2.75(−3.82, −1.69)	<0.01	−2.95(−4.13, −1.77)	<0.01

^a^Age at baseline and gender were adjusted. ^b^Age at baseline, gender, survey year, college, BMI, and vision were adjusted.

### Association between physical fitness level and four aspects of mental health scores

a) Association between physical fitness levels and physical symptom scores.

**Figure 2:**
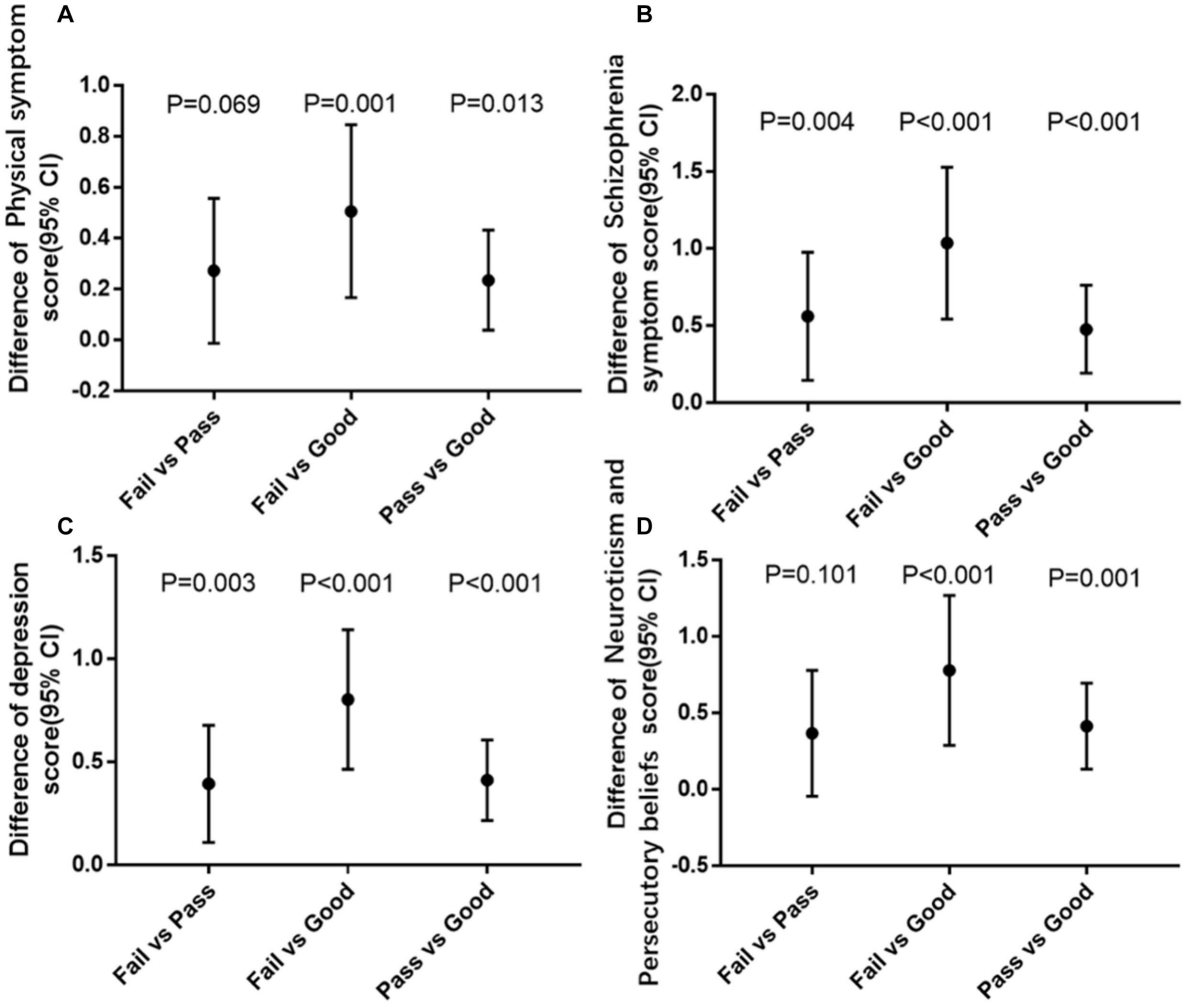
The association between four different symptoms of mental health and physical fitness levels. Age at baseline, gender, survey year, college, BMI, and vision were adjusted. CI Confidence interval, P Significance level. **(A)** Association between physical fitness levels and physical symptom scores. **(B)** Association between physical fitness levels and schizophrenia symptom. **(C)** Association between physical fitness levels and depression. **(D)** Association between physical fitness levels and neuroticism and persecutory beliefs.

In the multivariable-adjusted model (Table 3), participants with a good level of physical fitness (*β* = −0.53; 95% CI: −0.84, −0.22; *p* < 0.01) had a significantly lower risk of somatization (physical symptom) disorders, compared to the lowest level of physical fitness. Each increased score of physical fitness was associated with 0.02 lower scores of somatization disorders, with significant inverse associations also observed for risk of dementia (95% CI: −0.03, −0.01; *p* < 0.01; Supplementary Table S1). Besides, it is worth noting that students who failed the physical fitness tests had significantly higher physical symptom scores compared to those who achieved a superior grade on the physical fitness tests (*p* < 0.01). However, there was not a significant difference in physical symptom disorders between the failed group and the group who merely passed (Figure 2A).

- b) Association between physical fitness levels and schizophrenia symptom.

**Table 3:** Linear regression results of the levels of physical fitness and four different symptoms scores among the college students.

Levels of physical fitness	No. of participants	Model 1^a^		Model 2^b^	
		β (95%CI)	*p*-value	β (95%CI)	*p*-value
Somatization					
Failed	532	Reference		Reference	
Passed	5,142	−0.19(−0.42, 0.05)	0.12	−0.29(−0.55, −0.03)	0.03
Good	1,050	−0.41(−0.69, −0.14)	<0.01	−0.53(−0.84, −0.22)	<0.01
Schizophrenia					
Failed	532	Reference		Reference	
Passed	5,142	−0.54(−0.88, −0.20)	<0.01	−0.59(−0.97, −0.21)	<0.01
Good	1,050	−1.06(−1.46, −0.66)	<0.01	−1.11(−1.56, −0.66)	<0.01
Depression					
Failed	532	Reference		Reference	
Passed	5,142	−0.40(−0.63, −0.16)	<0.01	−0.43(−0.68, −0.17)	<0.01
Good	1,050	−0.85(−1.13, −0.58)	<0.01	−0.87(−1.18, −0.57)	<0.01
Neuroticism					
Failed	532	Reference		Reference	
Passed	5,142	−0.35(−0.68, −0.01)	0.04	−0.40(−0.76, −0.03)	0.03
Good	1,050	−0.83(−1.22, −0.44)	<0.01	−0.88(−1.31, −0.44)	<0.01

^a^Age at baseline and gender were adjusted. ^b^Age at baseline, gender, survey year, college, BMI, and vision were adjusted.

When moving to the schizophrenia symptom aspect, compared with individual with the failed level, the differences in schizophrenia symptoms among the passed and good groups were and scores were −0.59 (95% CI: −0.97, −0.21; *p* < 0.01) and − 1.11 (95% CI: −1.56, −0.66; *p* < 0.01), respectively (Table 2). The negative correlation between physical fitness level and schizophrenia symptoms (*β* = −0.04; 95% CI: −0.05, −0.03; *p* < 0.01) was revealed in Supplementary Table S1. Significant differences in schizophrenia symptoms existed among the three groups (Figure 2B).

- c) Association between physical fitness levels and depression.

In the depression aspect, compared with the failed group, differences in depression aspects were −0.43 scores (95% CI: −0.68, −0.17; *p* < 0.01) for passed group and −0.87 scores (95% CI: −1.18, −0.57; *p* < 0.01) for good group in the fully adjusted model. In addition, a negative relationship between physical fitness level and depression was observed, with per score increase in physical fitness test associated with a 0.04 score (95% CI: −0.03 to −0.02; *p* < 0.01) decline in depression scores (Supplementary Table S1). Furthermore, we found significant differences among the three students’ physical fitness level pairs of fail vs. pass, fail vs. good, and pass vs. good. (Figure 2C)

- d) Association between physical fitness levels and neuroticism and persecutory beliefs.

In neuroticism and persecutory beliefs aspects, students with failed physical fitness level got higher points in neuroticism and persecutory beliefs scores than those with passed (*β* = −0.40; 95%CI: −0.76 to −0.03; *p* = 0.03) or good (*β* = −0.88; 95%CI: −1.31, −0.44; *p* < 0.01) physical fitness level in the multivariable-adjusted model (Table 3). Additionally, the inverse linear association existed between physical fitness level and the score of neuroticism and persecutory beliefs (*β* = −0.03; 95%CI: −0.04, −0.02; *p <* 0.01). While differences were revealed between fail vs. good, and pass vs. good in Figure 2D, the difference was not significant between failed and passed groups.

In the comprehensively adjusted models, we noted a consistent association between levels of physical fitness and UPI scores across gender subgroups, as detailed in Supplementary Table S2. Similarly, the relationship between physical fitness levels and four facets of mental health scores did not differ significantly by gender, as shown in Supplementary Table S3. Furthermore, our analyses revealed no evidence of interaction between gender and physical fitness levels in their impact on mental health outcomes.

## Discussion

In this large-sample cross-sectional study, we found college students with higher levels of physical fitness were significantly associated with lower risks of mental disorders, including physical symptoms, schizophrenia, depression and neuroticism, and persecutory beliefs aspects. Among participants with varying levels of physical fitness, a significant, inverse association was observed between the emerging low-risk mental disorders. Whereas, in the physical symptom and neuroticism, and persecutory beliefs aspects, there was no significant difference between passed and failed groups. The psychological situations of students with comparatively low physical fitness levels should be considered. The association between physical fitness and mental health situations was independent of other potential confounders.

The association between physical fitness and mental health was partly conducted in previous studies. A meta-analysis assessing the association between cardiovascular fitness and the onset of psychological disorders illustrated that those with general or lower cardiovascular levels increased 23, 47% greater risk of experiencing mental disorders (17, 18), existing a significant dose–response relationship (19). Previous investigations suggested the potential benefits of healthy cardiovascular fitness in lowering the risk of psychological disorders, which was similar to the findings in the current study, while the evidence was limited in sample size, and just focused on cardiovascular fitness rather than overall physical fitness. Our study adds new evidence to this field that a higher overall physical fitness level is associated with a lower risk of overall psychological disorders among Chinese young adults. In addition, compared with peers, individuals with a higher level of physical fitness were commonly at lower risks of suffering from anxiety (20), depression (21), and hyperactivity inattention (22). In Nyberg J’s investigations (23), individuals with lower physical fitness were at 44% higher risk of schizophrenia problems. Similar results were also shown in our study. Compared with the failed group, scores of individuals with passed or good physical fitness levels declined 0.59 and 1.11 in schizophrenia symptom, respectively. Our investigation offered further evidence of the significant inverse associations between levels of physical fitness and the risks of disorders in physical symptoms, depression and neuroticism, and persecutory beliefs aspects.

It is noteworthy that our analysis revealed no significant difference in both physical and neurotic symptoms between students who marginally passed and those who failed the physical fitness test. This observation suggests that merely got the pass level in the fitness test does not suffice to confer mental health benefits among college students. The magnitude of the psychological health benefits seemed to depend on the level of physical fitness. These findings align with previous research indicating that the psychological health advantages associated with physical fitness are contingent upon surpassing a certain threshold of fitness. Several studies have demonstrated a dose–response relationship between levels of physical fitness and health outcomes. A meta-analysis evaluated the dose–response of cardiorespiratory fitness and stroke incidence, with risks of stroke declining by 15% for each increased unit of METs (24). Besides, findings from a cross-sectional study of 5,451 men and 1,277 women showed an inverse graded dose–response relationship between maximum cardiorespiratory fitness and depressive symptomatology (25). Hence, we speculated that individuals with low or medium levels of physical fitness might need to improve their fitness to generate more positive health outcomes. Moreover, the psychological status of individuals with low or medium levels of physical fitness should not be ignored, especially regarding physical symptoms and neurotic aspects.

The biologically plausible mechanism existed in a correlation between physical fitness and mental health situations. A high level of physical fitness achieved by regular physical activities could act as a buffer to reduce stress reactivity and prevent other metabolic results of stress-related events (26, 27). This was achieved by adjusting neuroendocrine and physiological responses (28), minimizing undue inflammation (29), and promoting neural plasticity (30). The improvement of aerobic fitness could alter hormonal stress-responsive systems and promote immune function, finally decreasing the adverse neurobiological effects caused by stressors, and stimulating the self-protection mechanism (28, 31). Furthermore, stable physical fitness situations and regular exercise habits could induce structural, cellular, or molecular changes in brains [such as hippocampal volume (32), neurotrophic factor (BDNF) (33), growth factor cascades (34)], generally enhancing neuroplasticity, benefiting the brain function and reducing the risk of psychological disorders, such as depression (30, 35).

Our study has some notable strengths. First, the major strength of our study was one of the large sample cross-sectional surveys, illustrating the positive association between the level of physical fitness and mental health situation among Chinese college students. Second, the whole investigation followed a strict quality data collection to ensure the results of the study’s robustness, validity, and reliability. The collection of physical fitness data utilized the objective measurements done by the trained staff. The standards for college students’ physique tests are reliable and specifically tailored toward students in different school grades. However, there are several certain limitations to our study. First, there were existing unmeasured factors that related to mental situations, such as dietary intake information, daily movement patterns (covering daily physical activities, sedentary behaviors, and sleep durations), alcohol and cigarette consumption, students’ annual family gross income, and household register. Due to security and privacy reasons, the administration staff of this college refused to release information regarding students’ family income and household registrations. The residual confounding unmeasured might exist in our cross-sectional study. Second, UPI cannot be considered a tool for diagnosing mental disorders. Third, these cross-sectional associations do not indicate causality, and future investigations are still warranted. Forth, we just recruited Chinese college students, thus the current findings in our study might not be generalizable to other groups with different cultural backgrounds.

## Conclusion

In general, we found robust evidence that the level of physical fitness was related to mental health and sleep situations among college students. Moreover, individuals with a low or medium level of physical fitness might face similar risks in the physical symptom and neurotic symptoms aspects. Therefore, the psychological situations of students with low physical fitness level should also be considered.

## Data availability statement

The raw data supporting the conclusions of this article will be made available by the authors, without undue reservation.

## Ethics statement

The studies involving humans were approved by the Medical Ethics Committee, Department of Psychological and Behavioral Sciences, Zhejiang University. The studies were conducted in accordance with the local legislation and institutional requirements. Written informed consent for participation in this study was provided by the participants’ legal guardians/next of kin.

## Author contributions

YHo: Writing – original draft, Writing – review & editing. JS: Writing – original draft, Writing – review & editing. YHu: Writing – original draft, Writing – review & editing. YG: Writing – original draft, Writing – review & editing. ZB: Writing – original draft, Writing – review & editing. YC: Writing – original draft, Writing – review & editing. SH: Writing – original draft, Writing – review & editing.
