# Supplementary material for: The association between physical fitness and mental health among college students: a cross-sectional study
Source: Front Public Health. 2024 Jul 16;12:1384035. doi: 10.3389/fpubh.2024.1384035 (PMC11286419; doi:10.3389/fpubh.2024.1384035)
Supplement: Supplementary file 1 [file Table_1.docx]

**Supplementary Table 1. Linear regression results of the physical fitness scores and UPI scores among the college students**

| **Fitness scores** | **Model 1^a^** | | **Model 2^b^** | |
| --- | --- | --- | --- | --- |
|  | **β (95%CI)** | **p-value** | **β (95%CI)** | **p-value** |
| **total scores** | -0.09(-0.12, -0.06) | <0.01 | -0.11(-0.14, -0.07) | <0.01 |
| **somatization** | -0.01(-0.02, -0.01) | <0.01 | -0.02(-0.03, -0.01) | <0.01 |
| **schizophrenia** | -0.03(-0.04, -0.02) | <0.01 | -0.04(-0.05, -0.03) | <0.01 |
| **depression** | -0.03(-0.03, -0.02) | <0.01 | -0.03(-0.04, -0.02) | <0.01 |
| **neuroticism** | -0.03(-0.04, -0.02) | <0.01 | -0.03(-0.04, -0.02) | <0.01 |

a: Age at baseline and gender were adjusted

b: Age at baseline, gender, survey year, college, BMI, and vision were adjusted.

**Supplementary Table 2. Linear regression results of the levels of physical fitness and UPI scores among the college students**

| **levels of physical fitness** | **Male** | | **Female** | | **P-interaction** |
| --- | --- | --- | --- | --- | --- |
|  | **β (95%CI)** | **p-value** | **β (95%CI)** | **p-value** |  |
| Failed | Reference | | Reference | | 0.33 |
| Passed | -1.43(-2.50, -0.37) | <0.01 | -1.03(-3.67, 1.62) | 0.45 |  |
| Good | -2.45(-3.80, -1.12) | <0.01 | -3.51(-6.35, -0.68) | 0.02 |  |

Age at baseline, gender, survey year, college, BMI, and vision were adjusted.

**Supplementary Table 3. Linear regression results of the levels of physical fitness and four different syptoms scores among the college students**

| **levels of physical fitness** | **Male** | | **Female** | | **P-interaction** |
| --- | --- | --- | --- | --- | --- |
|  | **β (95%CI)** | **p-value** | **β (95%CI)** | **p-value** |  |
| **somatization** |  | |  | | 0.72 |
| Failed | Reference | | Reference | |  |
| Passed | -0.30(-0.57, -0.02) | 0.04 | -0.22(-0.92, 0.47) | 0.53 |  |
| Good | -0.50(-0.85, -0.16) | <0.01 | -0.64(-1.39, 0.11) | 0.09 |  |
| **schizophrenia** |  |  |  |  | 0.24 |
| Failed | Reference | | Reference | |  |
| Passed | -0.59(-0.99, -0.19) | <0.01 | -0.40(-1.42, 0.61) | 0.44 |  |
| Good | -0.88(-1.39, -0.38) | <0.01 | -1.37(-2.45, -0.28) | 0.01 |  |
| **depression** |  |  |  |  | 0.15 |
| Failed | Reference | | Reference | |  |
| Passed | -0.42(-0.69, -0.15) | <0.01 | -0.33(-1.03, 0.36) | 0.34 |  |
| Good | -0.70(-1.05, -0.36) | <0.01 | -1.05(-1.80, -0.31) | <0.01 |  |
| **neuroticism** |  |  |  |  |  |
| Failed | Reference | | Reference | | 0.64 |
| Passed | -0.40(-0.80, -0.01) | 0.04 | -0.15(-1.11, 0.82) | 0.77 |  |
| Good | -0.76(-1.26, -0.26) | <0.01 | -0.87(-1.90, 0.16) | 0.10 |  |

bAge at baseline, gender, survey year, college, BMI, and vision were adjusted.
